# Toxicological and Biochemical Description of Synergism of *Beauveria bassiana* and Emamectin Benzoate against *Megalurothrips usitatus* (Bagrall)

**DOI:** 10.3390/jof8090916

**Published:** 2022-08-29

**Authors:** Youdan Zhang, Xiaochen Zhang, Qingheng Tian, Shaukat Ali, Liangde Tang, Jianhui Wu

**Affiliations:** 1 Key Laboratory of Bio-Pesticide Innovation and Application, Engineering Research Centre of Biological Control, South China Agricultural University, Guangzhou 510642, China; 2 Engineering Research Center of Biological Control, Ministry of Education and Guangdong Province, South China Agricultural University, Guangzhou 510642, China; 3 Taiqian County Agriculture and Rural Affairs Bureau, Puyang 457600, China; 4 Key Laboratory of Green Pesticide and Agricultural Bioengineering, Ministry of Education, Guizhou University, Guiyang 550025, China

**Keywords:** biopesticides, *Beauveria bassiana*, synergism, emamectin benzoate, *Megalurothrips usitatus*

## Abstract

The prophylactic application of synthetic insecticides to manage *Megalurothrips usitatus* (Bagrall) has resulted in insecticide resistance and negative impacts upon natural ecosystems. This has driven the need for developing alternative pest control strategies. In the present study, we investigated the synergistic interaction between the entomopathogenic fungus *Beauveria bassiana* and the insecticide emamectin benzoate on *M. usitatus*. The results of our research exhibited that higher doses of emamectin benzoate inhibited the germination rate and colony growth of *B. bassiana*. The percentage of *M. usitatus* mortality following *B. bassiana* and emamectin benzoate treatment indicated a dose–mortality effect. All concentrations of emamectin benzoate combined with different concentrations of *B. bassiana* demonstrated a synergistic effect five days post-treatment. When *B. bassiana* and emamectin benzoate were applied alone or in combination, antioxidant enzyme activities, including acetylcholinesterase, catalase, superoxide dismutase, and peroxidase, were significantly lower in *M. usiatus* than in the controls at the end of the experimental period. The findings of our study confirm the synergistic effect of *B. bassiana* and emamectin benzoate on *M. usitatus*, as well as the biochemical process that might be involved in the regulation of the synergistic effect.

## 1. Introduction

In China, *Megalurothrips usitatus* (Bagnall) (Thysanoptera: Thripidae) is a major pest of cowpea which attacks the leaves, flowers, and pods of cowpea, resulting in lower yields and quality [1,2,3]. Currently, the control measures of *M. usitatus* mainly rely on chemical pesticides. However, pesticides can be unsuccessful in suppressing thrips species because of their small size, cryptic nature, short developmental time, high fecundity, high pupation rate, and adult migratory ability [4,5]. Overuse of chemicals can also lead to harmful risks of pesticide resistance [6] and pesticide residues [7]. In an effort to develop environmentally friendly control measures, entomopathogenic fungi have become one of the most critical tools in the biological control of invertebrate pests due to their strong pathogenicity, broad host range, and low environmental impact [8,9].

For more than a century, entomopathogenic fungi have been considered potential biological control agents for different types of insects [10]. Attributes such as complex type of metabolism, diverse species range, and being safe for humans and non-target organisms make them promising alternatives over other tactics for pest control [11]. Among these fungi, *B. bassiana* has gained widespread attention for its broad-spectrum insecticidal activity for thrips management under laboratory and field conditions [12]. Furthermore, *B. bassiana* possesses many insecticidal advantages such as cuticle penetration, natural contact, and oral infection, cuticle-degrading enzyme secretion, and secondary metabolite production [13]. However, the field application of *B. bassiana* and other entomopathogenic fungi has received little attention because of a longer time interval required post application for insect mortality. Recently, many studies have investigated different methods to increase the virulence of these fungi such as the expression of growth modulating factors, expression of Bt toxin in *B. bassiana*, joint application with natural enemies, synthetic oils, bacterial metabolite avermectins, and its use with other biopesticides. [14,15].

Emamectin benzoate is a biological insecticide with a macrocyclic lactone structure. It is a highly effective semisynthetic derivative antibiotic insecticide derived from avermectin B1 fermentation [16]. Emamectin benzoate has broad-spectrum, ultra-high efficiency, low residues, and improved thermal stability compared to avermectin. It also has a 1–3-fold higher insecticidal activity when compared with avermectin [17]. Emamectin benzoate is effective against numerous insect pests, including lepidopteran insect larvae. It is also non-toxic to beneficial insects, making it an excellent candidate for use as an integrated insect pest control agent [18,19,20]. In this study, we hypothesized that the interaction of *B. bassiana* and emamectin benzoate could have a stable insecticidal impact at low concentrations, making a potential combination treatment for *M. usitatus* control [21]. Insects have a well-established defense mechanism against insecticides and natural pathogens, consisting of a wide range of enzyme systems. Detoxifying enzymes (acetylcholinesterase and glutathione-S-transferase) and antioxidant enzymes (superoxide dismutase, catalase, and peroxidase) are primarily responsible for herbivore defense against external challenges such as pathogens and chemicals [22,23]. Glutathione-S-transferase (GST) plays a key role in detoxification and cellular defenses by conjugating reduced glutathione to the electrophilic centers of foreign xenobiotics [24]. Acetylcholinesterase (AchE) is a key enzyme involved in the hydrolysis of acetylcholine in the nervous system of insects, whose activity can be affected by exogenous xenobiotics and fungal secondary metabolites [25]. Insect antioxidant enzymes superoxide dismutase (SOD), catalase (CAT), and peroxidase (POD) are known to be upregulated in response to xenobiotics threats [26]. This enzyme cascade protects insects from microbial infection by melanization of hemocytes attached to parasite surface [27]. In previous studies, it has been documented that a significant change in the activities of enzymes occurs against fungal infection and insecticidal poisoning [28]. To date, some studies have been conducted to understand the complex changes in enzyme profiles after the combined application of pathogens and insecticides against a variety of invertebrate pests [23]. However, the effects of synergistic action between *B. bassiana* and emamectin benzoate on the production of detoxifying and antioxidant enzymes by *M. usitatus* have not yet been reported.

The main aims of our work were to (a) study the pathogenic potential of *B. bassiana* isolate SB063 against *M. usitatus*; (b) determine the effect of emamectin benzoate on the biological characteristics (conidial germination and colony diameter) of *B. bassiana*; and (c) investigate the efficiency of various emamectin benzoate concentrations in combination with different conidial concentrations of *B. bassiana* against *M. usitatus*. The overall objective was to establish a scientific basis for the management of *M. usitatus* synergistically using a combination of *B. bassiana* and emamectin benzoate. 

## 2. Materials and Methods

### 2.1. Chemicals and Reagents

Emamectin benzoate (with a purity of 72.7%) was provided by Jiangsu Jinghong Chemical Co., Ltd., Yancheng, Jiangsu, China. The Bradford protein concentration assay kit was provided by Shanxi Biyuntian Biotechnology Com., Ltd., Xi’an Shanxi, China. All other chemicals and reagents used in this research work were of analytical grade. All chemicals were supplied by Shanghai Lingfeng Chemical Reagent Com., Ltd., Shanghai, China.

### 2.2. Insects

Adults of bean flower thrips were collected from the cowpea fields of Yongfa Town, Chengmai, Hainan, China. After collection, they were raised on bean pods in an artificial climate chamber at 26 °C, 70% relative humidity, and a 12 h:8 h (L:D) photoperiod as described by Espinosa et al. [29] and Du et al. [30].

### 2.3. Fungus

The isolate SB063 of *B. bassiana* used in the study was obtained from soil samples collected by Guangdong Provincial Key Lab of Biopesticides Innovation and Application, South China Agricultural University. For this study, fungal inoculum (1 × 10^8^ conidia/mL) was prepared using a previously prescribed method [10]. Serial dilutions were prepared by using different conidial concentrations with deionized H_2_O containing 0.05% Tween-80 solution.

*B. bassiana* isolate SB063 was cultured for 10 days on potato dextrose agar (PDA) plates under laboratory conditions. Conidial spores were harvested (by scraping the mycelia’s surface) using sterile cell scrapers and placing them in sterile deionized H_2_O (in 0.05% Tween-80). A hemocytometer was used to count the conidia (Qian Y. Glass Inst. Co., Ltd., Guangzhou, China) under a compound microscope (Ningbo Shunning Inst. Co., Ltd., Ningbo, China) at 40× magnification. The serial dilution approach was used to form lower concentrations (1 × 10^4^, 1 × 10^5^, 1 × 10^6^ and 1 × 10^7^ conidia/mL) for use in the virulence bioassays.

### 2.4. Emamectin Benzoate

Emamectin benzoate powder was dissolved in acetone to prepare a stock solution of emamectin benzoate (5.0 mg/L). The serial dilution method was again used to prepare lower doses of emamectin benzoate (5.0, 2.5, 1.25, 0.625, and 0.3125 mg/L) for subsequent experiments.

### 2.5. Emamectin Benzoate Impact on the Biological Features of Beauveria bassiana

Five different concentrations of emamectin benzoate (0.3125, 0.625, 1.25, 2.5, and 5.0 mg/L) were added to sterilized 50 mL Sabouraud dextrose broth (SDB), followed by *B. bassiana* conidial suspension (1 × 10^6^ conidia/mL; 2 mL) inoculation. Culture medium with only sterile H_2_O was used as a control treatment. The cultures were incubated at 180 rpm and 25 ± 2 °C for three days. After each treatment, samples of fungal cultures (1 mL) were examined using a microscope from Ningbo Shunning Inst. Co., Ltd., Guangzhou, China, to calculate total conidial germination using the methodology of Wu et al. [31]. The experiment was repeated three times, each with a new conidial suspension.

The impact of emamectin benzoate on radial growth of *B. bassiana* was examined by using five concentrations, i.e., 0.3125, 0.625, 1.25, 2.5, and 5.0 mg/L, of emamectin benzoate on PDA plates (10 mL PDA medium was placed into 9 cm Petri dishes). Mycelial plugs (2 cm diameter) of *B. bassiana* were inoculated onto the PDA plates followed by incubation at 26 °C, 85% relative humidity, and a 16:8 h light: dark photoperiod, for a period of seven days. The colony diameters were measured on a daily basis until day 7 using the methodology of Wu et al. [28].

### 2.6. Bioassay Studies

#### 2.6.1. Bioassay 1: *Beauveria bassiana* Isolate SB063 Efficacy against *Megalurothrips usitatus*

The effectiveness of *B. bassiana* against *M. usitatus* was investigated using the centrifuge tube residual method. Disposable plastic petri plates were soaked for 2 h in 1 × 10^4^, 1 × 10^5^, 1 × 10^6^, 1 × 10^7^, and 1 × 10^8^ conidia/mL, then air-dried. For each treatment, 20 mature female *M. usitatus* and the pods (1 cm) were used, and the experimental setup was repeated three times. For control, disposable plastic petri plates were soaked in ddH_2_O. Following treatment, plastic wrap was used to seal the disposable plastic Petri dishes. *M. usitatus* female mortality was checked daily for the following 7 days. The disposable plastic Petri dishes were incubated at a specific temperature (26 °C) and humidity (85%) with 16:8 h light: dark) photoperiod as outlined by Du et al. [27].

#### 2.6.2. Bioassay 2: Efficacy of Emamectin Benzoate against *Megalurothrips usitatus*

Impact of five different emamectin benzoate concentrations (0.3125, 0.625, 1.25, 2.5, and 5.0 mg/L) were examined under laboratory conditions against *M. usitatus*. Each disposable plastic Petri plate with pods (1 cm) was soaked for 2 h in various concentrations of emamectin benzoate and then dried under sterilized conditions. As a control, we utilized bean pods and disposable plastic Petri plates soaked in ddH_2_O. For each treatment, *M. usitatus* mature females (*n* = 20) were used, and the whole experiment was repeated three times. *M. usitatus* adult females were placed into a disposable petri dish and the bean pods were treated with the same concentration. Plastic wrap was used to seal the disposable plastic Petri dishes. *M. usitatus* female mortality was examined daily over the following 5 days. The disposable plastic petri plates were incubated at 26 °C, 85% (R.H.) and a photoperiod of 16:8 h light: dark.

#### 2.6.3. Bioassay 3: Efficacy of *Beauveria bassiana* and Emamectin Benzoate as Single or Combined Treatment Options against *Megalurothrips usitatus*

The effects of *B. bassiana* and emamectin benzoate on adult female *M. usitatus* were investigated separately or in combination using the centrifuge tube residual method as described in bioassays 1 and 2 above.

### 2.7. Megalurothrips Usitatus Enzyme Activity in Response to Single and Combined Treaments with Beauveria bassiana and Emamectin Benzoate

#### 2.7.1. Treatment of Insects and Sample Preparation

Female *M. usitatus* adults (*n* = 60) were treated with *B. bassiana* (1 × 10^6^ conidia/mL), emamectin benzoate (0.3125 mg/L), or a combination of both: B. bassiana (1 × 10^6^ conidia/mL) and emamectin benzoate (0.3125 mg/L), using the centrifuge tube residual method as described earlier. For control, disposable plastic Petri plates were soaked in ddH_2_O. The treated insects were collected 3–5 days post-treatment. For enzyme assays, the collected insects were homogenized in ice-cold potassium phosphate buffer (0.05 M) as described by Ali et al. [10].

#### 2.7.2. Total Protein Assay

The Bradford method was used to measure the total protein concentration in the insect samples [32].

#### 2.7.3. Enzyme Activity Assays

To determine acetylcholinesterase (AChE) activity in *M. usitatus*, the methodology of Ellman et al. [33] was used. Variations in the reaction mixture 50 μL solution of the sample having 100 μL of 45 μM 5-5-dithiobis-(2-nitrobenzoic acid), acetylthiocholine iodide (100 μL) and sodium phosphate buffer (90 μL) were noticed at 405 nm absorbance for 40 min. The enzyme activity was calculated as the change in (per minute/mg) protein absorbance.

The glutathione-S-transferase (GSTs) assays were conducted according to Habig et al. [34]. The samples were incubated at 25 °C for five minutes in sodium-phosphate buffer (0.1 M) with a pH of 6.5 having 1 mM glutathione (1 mM), DNCB (1 mM), and sample (20 μL). DNCB solution was used in acetone to initiate the reaction. The amount of 5-(2,4’-dinitrophenyl) glutathione evolved during the reaction was assessed by using a spectrophotometer (at 340 nm wavelength). One unit of enzyme conjugated CDNB at the rate of 10.0 nmol with reduced glutathione per minute.

The method of Beauchamp et al. [35] was used to conduct the superoxide dismutase (SOD) assay. The quantity of enzyme (SOD) required for inhibition of fifty percent nitro blue tetrazolium reduction reaction (per mg protein) at 560 nm was considered as one unit enzyme activity. 

A spectrophotometric study of hydrogen peroxide (H_2_O_2_) decomposition at 240 nm was used to assess catalase (CAT) activity [36]. The enzyme quantity required to decompose 1 mmol H_2_O_2_ per minute at an initial H_2_O_2_ concentration (30 mM, at pH 7.0) and temperature (25 °C) was defined as one unit of catalase activity. 

The methodology of Shannon et al. [37] was used to conduct a peroxidase (POD) assay. Firstly, sample and potassium phosphate 14 mM buffer (0.1 mL) were added in the reaction mixture and incubated (20 °C, for 10 min) before being mixed through inversion. Variations in absorbance were recorded at a specific wavelength (420 nm).

### 2.8. Data Analysis

All data analysis was performed by using SPSS 19.0 Software [38]. Radial growth and germination data were analyzed by using ANOVA. Comparison of means was carried out by HSD test of Tukey with *p* < 0.05. 

Data regarding *M. usitatus* mortality in response to different concentrations of *B. bassiana* or emamectin benzoate was arcsine transformed and means were compared by analysis of variance (ANOVA-1) and significance of means was compared with Tukey’s HSD test at 5% level of significance whereas median lethal concentrations (LC_50_) were calculated by Probit analysis. Synergism levels between the *B. bassiana* and emamectin benzoate were calculated by using the following equations [35]. Initially, the corrected mortality over control was calculated by following equation:

$$Corrected\;Mortality\;\left(M\right)=\frac{\left(Mortality\;in\;response\;to\;treatment-Mortality\;in\;response\;to\;control\right)}{\left(1-Mortality\;in\;response\;to\;control\right)}$$

The expected mortality in response to different combined treatments of *B. bassiana* and emamectin benzoate as well as Chi square values to determine the kind of interaction were calculated through following equations:

*Expected mortality* (*M_e_*) = *M* + *M_B_* ∗ (1 − *M*)

where *M_e_* is the expected mortality for additive mortality; *M_A_*, *M_B_*, and *M_AB_* are the observed mortalities for *B. bassiana*, emamectin benzoate, and their combination, respectively. The significant differences between the observed and expected mortality were defined through significance of the chi square values calculated through following equation:

$$Chi\;square\;\left(\chi^{2}\right)=\frac{\left(M_{B}-M_{e}\right)*100*\left(M_{B}-M_{e}\right)}{\left(M_{e}\right)}$$

Then, *p*-values were looked up in the Chi square table for df = 1.

If the *M_AB_* was significantly lower than *M_e_* (when the calculated *χ*^2^ was lower than expected *χ*^2^ value observed from the Chi square table), it meant antagonism. If the *M_AB_* was significantly higher than *M_e_* (when the calculated *χ*^2^ was higher than expected *χ*^2^ value observed from the Chi square table), it meant synergism. Otherwise, the mortality was additive [39]. 

## 3. Results

### 3.1. Effect of Emamectin Benzoate on the Biological Characteristics of Beauveria bassiana

**Figure 1:**
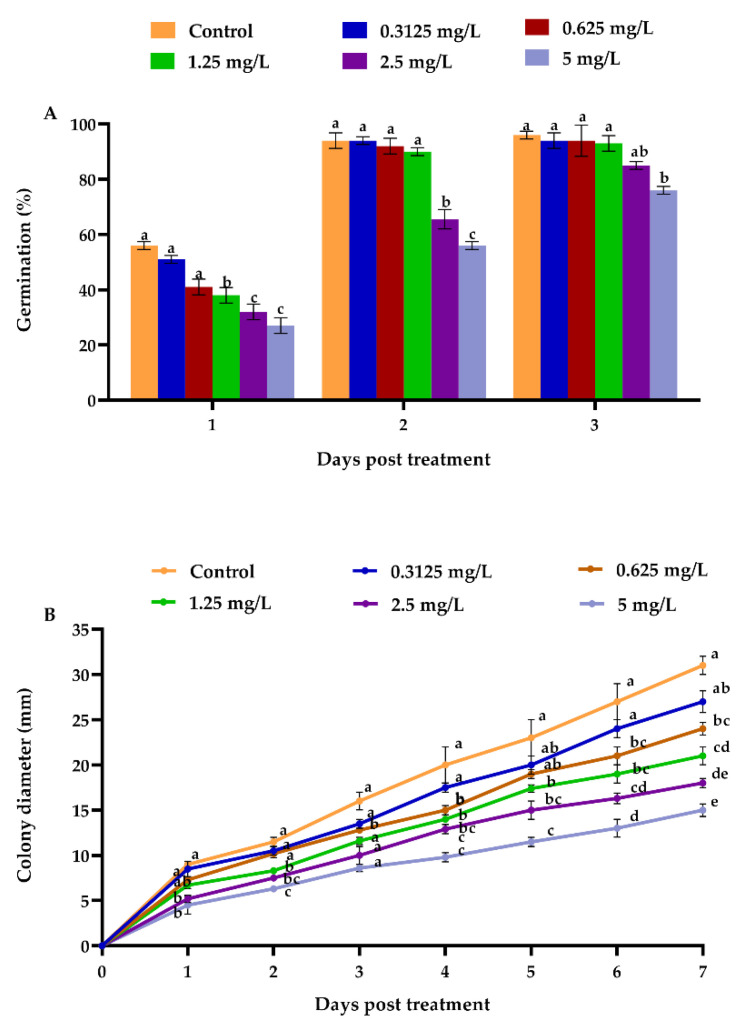
Emamectin benzoate effect on the biological features of *B. bassiana*. (**A**) Germination rate (%); (**B**) colony diameter (mm). Error bars indicate standard error of means based on three replicates. Bars with different letters indicate significant differences between treatments at various periods of time (Tukey’s test at 5% level of significance).

Our results demonstrated that different emamectin benzoate concentrations significantly affected the germination rate (%) of *B. bassiana* compared to the control at the end of experimental period (*F* = 13.22; df = 5, 12; *p* = 0.003) (Figure 1A). A reduction in germination rate (76%) was noticed at 5.00 mg/L emamectin benzoate concentration after three days of growth; however, the germination rate in the control was the highest (Figure 1A). When compared to the control, different emamectin benzoate concentrations had a significant effect on *B. bassiana* radial growth at the end of experimental period (*F* = 44.54; df = 5, 12; *p* < 0.001) (Figure 1B). When the concentration of emamectin benzoate was increased, the average colony diameter was reduced. The control had the highest radial growth (31 mm) after 7 days of growth, while the 5.00 mg/L emamectin benzoate treatment had the lowest colony diameter (averaging nearly 15 mm) (Figure 1B).

### 3.2. Bioassay Studies

#### 3.2.1. *Beauveria bassiana* Efficacy against *Megalurothrips usitatus*

**Figure 2:**
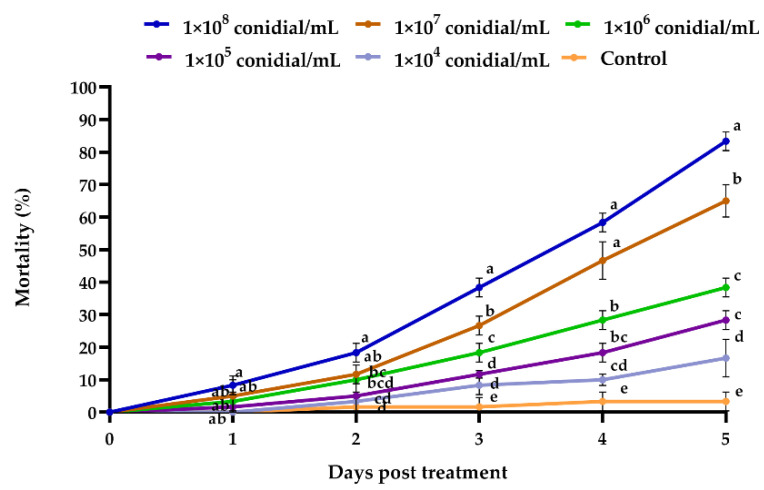
Mortality percentage of *M. usitatus* (*N* = 3) in response to *B. bassiana* applications, at various time intervals. The standard error of the means based on three replicates is indicated by the error bars. Bars with different letters were significantly different from one another at different days after treatment.

*M. usitatus* (adult females, *n* = 60) challenged by different conidial concentrations of *B. bassiana* had different mortality percentages (Figure 2). The conidia concentration with the highest mortality, at different days after fungal treatment was 1 × 10^8^ conidia/mL, with mean mortality of 83%. The LC_50_ of *B. bassiana* was 1.73 × 10^7^ conidia/mL seven days post-treatment.

#### 3.2.2. Emamectin Benzoate Efficacy towards *Megalurothrips usitatus*

**Figure 3:**
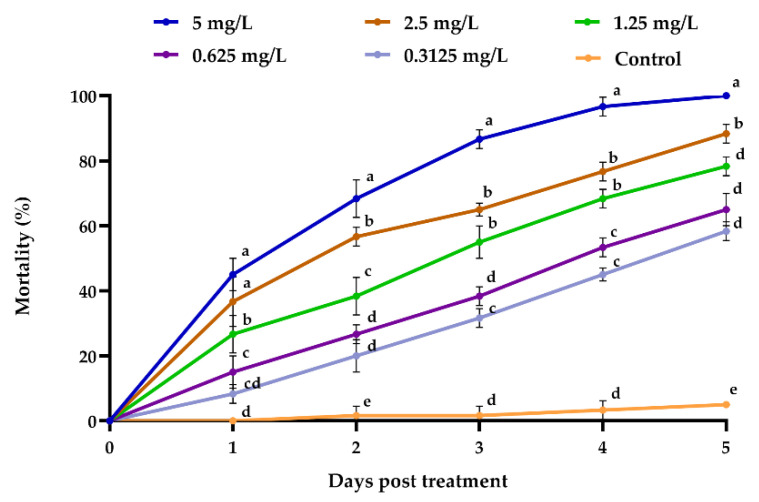
Mortality (%) of *M. usitatus* (*N* = 3) challenged with various emamectin benzoate concentrations at different time intervals. The standard error of the means (three replicates) is indicated by the error bars. Bars showing different letters were significantly different from one another on different days after treatment.

When compared to the control, differing emamectin benzoate concentrations demonstrated a significant difference in the mortality rate of *M. usitatus* at the end of experimental period (*F* = 201.15; df = 5, 12; *p* < 0.001). The death rates of *M. usitatus* treated with 5.0 mg/L emamectin benzoate and 2.50 mg/L emamectin benzoate after 5 days were 100% and 89%, respectively (Figure 3). The LC_50_ of emamectin benzoate was 0.285 mg/mL five days post-treatment.

#### 3.2.3. Efficacy of *Beauveria bassiana* and Emamectin Benzoate as Single or Combined Treatments against *Megalurothrips usitatus*

When *B. bassiana* and emamectin benzoate were used in combination, a significantly higher mortality was observed in *M. usitatus* than the control and their respective individual treatments (Table 1). A synergistic effect was observed when all concentrations of emamectin benzoate were combined to different *B. bassiana* conidial concentrations, four days post-treatment. Greater than 90% mortality was noticed after the application of different combinations, among which, emamectin benzoate and *B. bassiana* showed 100% mortality at 0.625 mg/L and *B. bassiana* at 1 × 10^7^ and 1 × 10^6^ conidia/mL, respectively.

### 3.3. Megalurothrips Usitatus Enzymatic Response to Individual or Combined Treatments of Beauveria bassiana and Emamectin Benzoate

**Figure 4:**
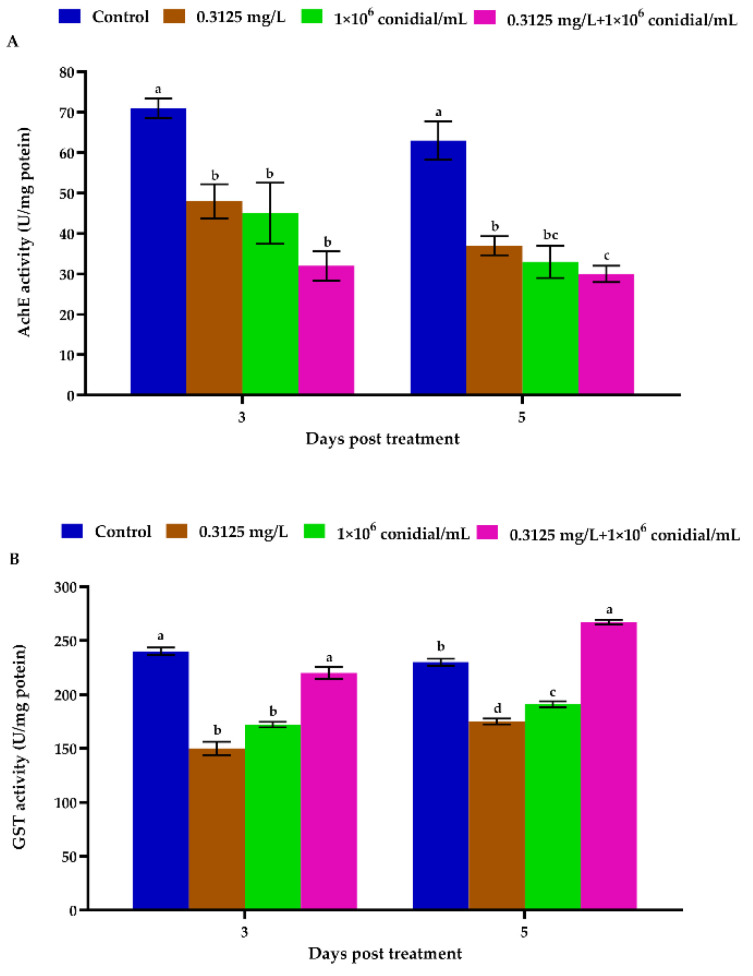
Acetylcholinesterase (AChE) and glutathione S-transferase (GST) activity (*N* = 9) of *M. usitatus* in response to single or joint treatments of *B. bassiana* and emamectin benzoate, 3 and 5 days of treatment. The activity of acetylcholinesterase (AChE) (**A**); activity of glutathione S-transferase (GST) (**B**). Error bars indicate standard error of means based on three replicates. Bars with different letters indicate significant differences between treatments at various periods of time (Tukey’s test at 5% level of significance).

Our results demonstrated a significant reduction in the activity of acetylcholinesterase (AChE) on day 3 (*F* = 22.41; df = 3, 8; *p* = 0.006) and 5 (*F* = 43.32; df = 3, 8; *p* = 0.002). Interestingly, acetylcholinesterase (AChE) activity was much lower at the end of the experiment than three days after treatment. On the fifth day, the glutathione S-transferase (GST) activity of *M. usitatus* of all treatments except the control was higher than that on the third day (Figure 4).

**Figure 5:**
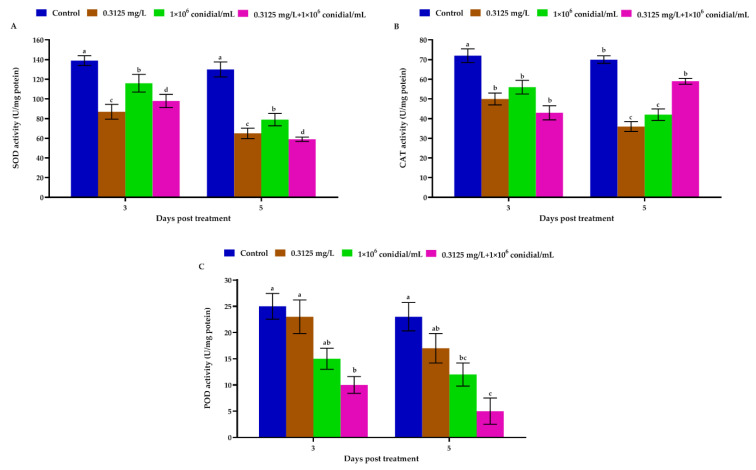
Antioxidant enzyme activities of *M. usitatus* (*N* = 9) in response to single or combined treatments of *B. bassiana* and emamectin benzoate) at 3 and 5 days post-treatment. (**A**) Activity of superoxide dismutase (SOD), (**B**) catalase (CAT), (**C**) and Peroxidase (POD). Error bars indicate standard error of means based on three replicates. Different letters on bars indicate significant differences among treatments at different periods of time (Tukey’s test at 5% level of significance).

Following exposure of *M. usitatus* adults to *B. bassiana* and emamectin benzoate, the SOD activities differed remarkably after 3 (*F* = 43.97; df = 3, 8; *p* = 0.006) and 5 days (*F* = 166.65; df = 3, 8; *p* < 0.001) post treatment (Figure 5A). The results revealed that SOD activities in relation to different treatments were significantly different from that of the control. On the 5th day post treatment, the lowest SOD activity was observed for *B. bassiana* + spinetoram treatment. The SOD activities observed for *B. bassiana* treatment were significantly higher than SOD activities in response to emamectin benzoate treatment (Figure 5A). 

As can be seen in Figure 5B, *M. usitatus* adults treated with individual or joint treatments of *B. bassiana* and emamectin benzoate showed significant differences for catalase (CAT) activity at different time intervals. The CAT activities in response to *B. bassiana* treatment were significantly higher than CAT activities in response to emamectin benzoate treatment. The CAT activities in response to *B. bassiana* + emamectin benzoate treatment were significantly lower than the control after 3 (*F* = 19.73; df = 3, 8; *p* = 0.07) and 5 days (*F* = 74.74; df = 3, 8; *p* = 0.001) post treatment.

The peroxidase (POD) activities of *M. usitatus* adults treated with individual or combined treatments of *B. bassiana* and emamectin benzoate showed significant differences at different time intervals. The POD activities in relation to changing treatments were considerably lower than the control after 3 (*F* = 10.87; df = 3, 8; *p* = 0.022) and 5 days (*F* = 14.56; df = 3, 8; *p* = 0.013) post treatment. On day 3 and 5 post treatment, POD activities in response to *B. bassiana* + emamectin benzoate treatment were significantly lower than the remaining treatments and control (Figure 5C).

## 4. Discussion

Understanding the effects of synthetic insecticides on growth, virulence, and enzyme activity is a prerequisite for development of integrated control tactics based on synthetic chemicals and entomopathogenic fungi [32]. However, just a few studies have explored the potential compatibility of *B. bassiana* with chemicals (from living organisms or plants) and some biomolecules with novel modes of action [31,39].

Our findings demonstrated that different emamectin benzoate concentrations caused a significant reduction in radial growth of *B. bassiana* compared to controls whereas germination (%) was not affected. Our findings are line with Wu et al. [31] and Wu et al. [12], who investigated the effects of matrine on *Akanthomyces attenuatus* growth and germination. Their results also revealed that the germination rate and colony growth of *Akanthomyces attenuatus* were inhibited by higher matrine concentrations. 

The virulence of an insect pathogen can vary against different developmental instars of its insect host [6]. The concentration–mortality response of *B. bassiana* isolate SB063 against *M. usitatus* showed a varied response. The concentration–mortality of *M. usitatus* caused to *B. bassiana* during this study was lower compared with the application of a different *B. bassiana* isolate indicated by Yang et al. [3]. Emamectin benzoate activity was also assessed against *M. usitatus*. Our mortality data showed that LC_50_ of emamectin benzoate against *M. usitatus* 5 days post inoculation was 0.285 mg/L which is different from the LC_50_ of emamectin benzoate against *Frankliniella occidentlis* reported by Cruces et al. [40]. The results of our study differ from those of other studies in part, possibly because of a different host population or different source of fungal isolation.

The combination of *B. bassiana* with emamectin benzoate had a substantial synergistic impact on *M. usitatus* (under laboratory conditions). Previous studies have used different models (calculation of synergism based on the fold increase in mortality, using Chi square relationship to evaluate the synergism, and mixing similar pest control agents) to evaluate the synergistic interactions between different pest control treatments [12,41,42,43]. The study by Xu et al. [44] indicated that the selection of these models for a mixture of chemicals and insect pathogenic fungi is inappropriate if the mortality is caused by both a pathogen and a chemical. However, the level of synergistic interaction in this study was additive (a higher mortality rate than expected). The observed synergism is related to the mode of action of both products. Emamectin benzoate is a chloride channel activator that stimulates high-affinity γ-amino butyric acid receptors and glutamate-gated chloride channels, ultimately disrupting nerve signals in arthropods. The insect larvae stop feeding after exposure and become irreversibly paralyzed, leading to death within 3 or 4 days [45]. *B. bassiana* infects its hosts through different processes such as cuticle penetration, natural contact, and oral infection, secretion of cuticle degrading enzymes, and production of secondary metabolites [13]. Initially, emamectin benzoate stressed the insects, making them more susceptible to fungal infection, resulting therefore in higher synergism between both pest control agents. 

Our results also indicated the biochemical and physiological impact of treating *M. usitatus* with a combination of *B. bassiana* and emamectin benzoate. The enzyme activities fluctuated significantly following *B. bassiana* and emamectin benzoate treatments (applied single or joint). Acetylcholinesterase (AChE), as a critical central nervous system enzyme of insects, plays a critical role in the acetylcholine hydrolysis. In the present work, *M. usitatus* AChE activities in response to various treatments were significantly reduced compared with the control. However, this indicates that decreased AChE activity is linked with a common site of action (within the insect host). *Beauverai bassiana* can produce a secondary metabolite named bassianolide (a cyclooligomer depsipeptide) which can affect acetylcholine receptors of insect muscles reducing the production of AChE [46]. Emmamectin benzoate is known to target insect acetylcholine (AChE) receptors which in turn affects AChE production [17]. The reduction in AChE activity of *M. usitatus* following *B. bassiana* application is in line with the findings of Zibaee et al. [28] who observed similar inhibition of AChE activity when *Beauveria bassiana* and its secondary metabolites were applied against sunn pest (*Eurygaster integriceps*).

Antioxidant enzymes, including CAT, SOD, and POD, are cellular system enzymes that reduce the damage of the bio-membrane by eliminating ROS [47]. In response to different treatments, SOD, CAT, and POD activities were higher after three days than five days post-treatment. Thus, the inhibition of these enzymes during the later period of the experiment suggests reduced ROS elimination with denaturation of various molecules within the insect body, which results in insect mortality.

## 5. Conclusions

Our results display a promising synergistic mechanism (between emamectin benzoate and *B. bassiana*) against *M. usitatus* since emamectin benzoate did not affect fungal conidial germination. The response of *M. usitatus* enzymes against single or combined treatments of *B. bassiana* and emamectin benzoate further clarified the biochemical nature of the synergistic effect. 

Based on the results of current and previous studies, we can hypothesize that the enzymatic defense of *M. usitatus* is activated by the assault of either emamectin benzoate or *B. bassiana* and that the combination of these two agents can overcome this defense strategy through a strong synergistic effect. The chemical basis of this strong synergistic effect is possibly related to the disturbance of the acetylcholine balance and changes in AChE activities of the thrips as both emamectin benzoate and *B. bassiana* can target insect acetylcholine (ACh) receptors which in turn effects AChE production

Such information is highly useful in designing integrated pest control programs against *M. usitatus*.

## Figures and Tables

**Table 1:** Mortality (%) of *M. usitatus* in response to single or combined application of *Beauveria bassiana* and emamectin benzoate treatments.

**Treatments**		***Megalurothrips usitatus* Mortality (%) at Different Time Intervals (d)**				
		**1**	**2**	**3**	**4**	**5**
**Emamectin benzoate (mg/L)**	**0.315**	8.33 ± 1.67 de	20 cd	31.67 ± 1.67 cd	45 ± 2.89 c	58.33 ± 4.41 c
	**0.625**	15 cd	26.67 ± 1.67 c	38.33 ± 1.67 c	53.33 ± 3.33 bc	65 ± 2.89 c
***Beauveria bassiana* (conidia/mL)**	**1 × 10^7^**	5 ± 2.89 e	11.67 ± 1.67 de	26.67 ± 1.67 de	46.67 ± 1.67 c	65 ± 2.89 c
	**1 × 10^6^**	3.33 ± 1.67 e	10 ± 2.89 de	18.33 ± 1.67 e	28.33 ± 3.33 d	38.33 ± 1.67 d
***Beauveria bassiana* (conidia/mL) + Emamectin benzoate (mg/L)**	**1 × 10^7^ + 0.625**	38.33 * ± 1.67 a	61.67 * ± 1.67 a	81.67 * ± 1.67 a	100 a *	100 a
		(19.25; 18.91)	(35.23; 19.85)	(54.78; 13.20)	(75.11;8.25)	(87.75; 1.71)
	**1 × 10^6^ + 0.625**	26.67 * ± 1.67 b	53.33 * ± 3.33 a	70 * ± 2.89 b	100 a *	100 a *
		(17.83; 4.38)	(34.00; 10.99)	(49.63; 8.36)	(66.55; 16.81)	(78.42; 5.94)
	**1 × 10^7^ + 0.3125**	30 * ± 2.89 ab	53.33 * ± 3.33 a	71.67 * ± 1.67 b	98.33 * ± 1.67 a	100a
		(12.91; 22.60)	(29.34; 19.62)	(49.89;9.50)	(70.67; 10.83)	(85.41; 2.49)
	**1 × 10^6^ + 0.3125**	23.33 * ± 1.67 bc	41.67 * ± 3.33 b	63.33 * ± 1.67 b	93.33 * ± 1.67 a	100 a *
		(11.38; 12.54)	(28.00; 6.67)	(44.19; 8.28)	(60.58; 17.70)	(74.30; 8.89)
**Control**	**0**	1.67 ± 1.67 e	3.33 ± 1.67 e	3.33 ± 1.67 f	5 e	6.67 ± 1.67 e
***F*; df; *p***		50.40; 8, 18 <0.001	82.42; 8, 18 <0.001	221.73; 8, 18 <0.001	293.79; 8, 18 <0.001	237.57; 8, 18 <0.001

The difference between the means (±SE) followed by various letters is significant (Tukey’s *p* < 0.05). The projected mortality and *χ*^2^-values are shown in brackets, respectively. * indicates a synergistic interaction.

## Data Availability

The raw data supporting the conclusions of this manuscript will be made available by the authors, without undue reservation, to any qualified researcher upon request.
